# Physicochemical properties and antioxidant activities of marula fruit (*Sclerocarya birrea* subsp. *Caffra*) steamed and boiled before juice extraction

**DOI:** 10.1002/fsn3.3423

**Published:** 2023-05-10

**Authors:** Mokoena Z. Dorothy, Terence N. Suinyuy, John Lubaale, Bamidele O. Peter

**Affiliations:** ^1^ School of Agricultural Science University of Mpumalanga Mbombela South Africa; ^2^ School of Environmental Science University of Mpumalanga Mbombela South Africa; ^3^ Department of Consumer and Food Sciences University of Pretoria Pretoria South Africa; ^4^ Department of Food Science and Technology University of Venda Thohoyandou South Africa

**Keywords:** extraction, juice, marula, processing, sensory

## Abstract

Marula fruit is one of the most underutilized fruits in South Africa, and it has been reported to contain a high amount of vitamin C which is regarded as the cheapest antioxidant. The fruit pulp is traditionally extracted and boiled into juice, a process that adversely affects the vitamin C and bioactive phenolic profile of the resulting juice. This study evaluated the effects of boiling and steaming on the physicochemical properties of marula fruit juice. The pH, percentage yield, total titratable acidity (TTA), total soluble solids (TSS), total phenolic content (TPC), radical scavenging capacity, and vitamin C content of the fruit juice were examined. The study also investigated the total carotene, color, and sensory properties of the fruit juice. The results showed that boiling and steaming significantly decreased the Vit C content of the juice (75.67 and 60.05 mg/100 g) compared to control sample (95.11 mg/100 g). The TPC, radical scavenging capacity, and total carotene content of the fruit juice increase because the heating processes softened the matrix of the fruit increasing the extractability of the phenolics and carotene content of the samples. The color of the marula fruit juice was increased by both boiling and steaming, while the sensory properties of the marula fruit juice extracted from steamed marula fruit had the highest scores in all the measured parameters. Steaming of marula fruit before juice extraction improved the nutritional composition, antioxidant activities, and sensory properties of marula fruit juice.

## Graphical abstract

This study showed the importance of processing on nutritional qualities of marula fruit juice. The boiling and steaming decrease the vitamin C content of the juice when compared to unprocessed marula fruit. Steaming of the fruit (Marula) before juicing increases the total phenolic content and antioxidant activities of the marula fruit juice. Also, the steaming improves the yield and the color of the juice. Steaming marula fruit before juice may be a better option than boiling it.

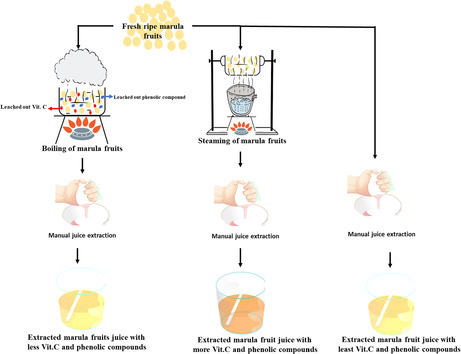

## 1 INTRODUCTION

Marula fruit (*Sclerocarya birrea* subsp. *Caffra)* is to a great extent the most utilized indigenous wild fruit for local rural communities of southern African communities (Sinthumule & Mashau, 2019). The fruits are consumed in their raw form or transformed into by‐products such as jams, beverages, concentrates, and the popular fermented alcoholic beverage known as Amarula (Dlamini, 2011; Schulze‐Kaysers et al., 2015). Marula fruit juice has been investigated for its exceptionally high quantity of vitamin C content, reported to be about 62–400 mg per 100 g in fresh juice (Hiwilepo‐van Hal et al., 2012). Due to its high vitamin C content, marula fruit could act as an alternative source of vitamin C for individuals seeking to variegate from market‐driven fruits such as citrus fruits, apples, grapes, and guabiroba fruit (Grutzmann Arcari et al., 2020; Sinthumule & Mashau, 2019).

Notwithstanding its higher content of vitamin C, marula fruit is also rich in bioactive phenolic compounds such as catechin, epicatechin, quercetin, myricetin, and proanthocyanidins (Hiwilepo‐van Hal et al., 2014; Mashau et al., 2022). The superior fatty acid and amino acid profile of the marula fruit among other fruits native to Africa has placed it among the essential fruits that can promote health and requires further investigation into the different varieties of the fruit (Kamanula et al., 2022; Mariod & Abdelwahab, 2012). Extracting juice from fruits requires different unit operations such as washing, boiling, steaming, peeling, juicing, sieving, pasteurization, bottling and capping, and storage (Renard & Maingonnat, 2012). It is worth noting that not all fruits undergo all these unit operations and as such, for the extraction of juice from marula fruits (Alehosseini et al., 2021), the local people boil the fruits before extraction of the juice as a way of increasing the recovery of juice from the fruits and enhancing the nutritional profile of the juice (Hiwilepo‐Van Hal et al., 2013; Shackleton & Shackleton, 2002).

Boiling whole fruits before extraction may have both positive and negative effects on the nutritional composition of such fruit juice (Ahmed & Eun, 2018). Boiling could result in the loss of water‐soluble vitamins (A and C) and other soluble phenolic compounds (anthocyanins, Hydrocinnamate, and tangeretin) (Yaman et al., 2021). Steaming of fruit before extraction of the juice may be more efficient than boiling since the fruits are not in contact with water which may prevent the water‐soluble phytochemicals and vitamins (Rossi et al., 2003; Youssef et al., 2002).

It was hypothesized that marula fruit steamed before extraction of the juice will be higher in nutritional composition than marula fruit boiled before the juice is extracted. Hence, this study is aimed at determining the physicochemical, antioxidant, and sensory properties of steamed and boiled marula fruit juice.

## 2 MATERIALS AND METHODS

Mature, ripe, and fresh Marula fruit (*Sclerocarya birrea* subsp. *Caffra*) was collected at three different locations (University of Mpumalanga, Valencial, and Mattafin (2021)) within Mbombela City, Mpumalanga, in a random sampling approach. Muslin cloths, beakers, knives, and translucent plastic bottles were used for the extraction of the marula juice. Analytical chemicals were purchased from Merck Chemicals in Johannesburg.

### 2.1 Sample preparation

Marula fruit upon collection was taken to the University laboratory, sorted, washed with distilled water, and divided into three different batches. Batch 1 was juiced after the removal of the peel, the second batch was boiled at 100°C for 5 min before juicing, and the last batch was steamed for 5 min before juicing. The marula fruit juice was extracted manually using clean hands and clean muslin cloths. The marula fruit juice was stored inside translucent 100 mL plastic bottles, pasteurized, and stored at refrigeration temperature (4°C) for further analysis.

### 2.2 Determination of pH, percentage yield, total soluble solids ( ^0^Brix), and total titratable acidity (TTA)

A pH meter (Hanna Instrument, Poroa de Varzim, Portugal) previously calibrated with buffer solutions (4 and 7) was used to determine the pH of the samples. The percentage yield was calculated using Equation 1 below. Total soluble solids were determined in °Brix using a handheld refractometer previously adjusted to zero with distilled water (Hanna Instruments, Italy). The prism of the refractometer was cleaned with distilled water after each analysis. The titratable acidity of the samples was determined according to the method of the AOAC (2005).

(1) $$\text{Percentage fruit yield}=\frac{\text{Amount of marula fruit juice extracted}\;\left(\mathrm{mL}\right)}{\text{Amount of marula fruits}\;\left(g\right)}\times 100$$

### 2.3 Ascorbic acid content (AA)

AOAC (2005) method was used to determine the ascorbic acid content of marula fruit juice samples. About 20 mL of the marula fruit juice samples was made up to 50 mL with oxalic acid (0.1 M) (metal chelator) and filtered. A pipette was used to dispense the filtrate (5 mL) into a beaker, where it was titrated with standardized 2,6‐dichlorophenol indophenol dye. When the titration ended, the colored solution turned from orange to pink. Three times, the process was carried out. The titer results were translated to mg of ascorbic acid per liter of sample marula fruit juice.

### 2.4 Total phenols (TPC) determination

Tezcan et al.'s (2009) modified method using the Folin–Ciocalteu was used to evaluate the total phenolic contents of marula fruit juice. About 300 μL of diluted marula fruit juice samples (1 mL of marula fruit juice samples: 100 mL of water) with methanol: water (6:4 (*v/v*)) were combined with 1.5 mL of the Folin–Ciocalteu reagent, which had been diluted five times, and 1.2 mL of 7.5% sodium carbonate. A Hitachi, Model 100‐20 spectrophotometer was used to test the mixture's absorbance at 760 nm after it had been allowed to stand for 60 min at room temperature. On a dry weight basis, total phenols were reported as mg catechin equivalents (μL CE/mg). The calibration curve using catechin as a standard has a line equation:

$$y=1.05\text{4x}–0.1682\;\left(R^{2}=.9988\right).$$

### 2.5 ABTS ^+^ radical scavenging activity determination

Using the method outlined by Awika et al. (2003), the ABTS^+^ radical scavenging activity of the marula fruit juice was determined. The reaction mixture was incubated for 30 minutes after the ABTS stock solution (2 mL of ABTS stock solution, 0.01 M) was added to 58 mL of phosphate buffer saline (pH 6.9) and had been incubated for 12 hours. At 734 nm, the absorbance was measured. Results were expressed as micromole Trolox equivalents per gram of material (mol TE/g) on a dry weight basis, using Trolox as the standard.

### 2.6 DPPH radical scavenging activity determination

Using the method outlined by Apea‐Bah et al. (2014), the DPPH radical scavenging activity of the marula fruit juice samples was determined. A 0.102 mM working solution was made from a 0.609 mM DPPH stock solution made in 80% (*v/v*) aqueous methanol. In a 96‐well plate, a 5 × dilution of the marula fruit juice mixture (10 μL) was combined with 190 μL of DPPH working solution and incubated for 1 h in the dark at a temperature of 15 ± 2°C. Using a microplate reader, the absorbance was measured at 570 nm (Multiskan FC, Thermo Fisher Scientific, Shanghai, China). Results were expressed as millimoles of Trolox equivalent per milligram of sample (mol TE/mg) on a dry weight basis using Trolox as the standard.

### 2.7 Total carotene determination

The spectrophotometric (Hitachi, Model 100‐20) method, as modified by Goula and Adamopoulos (2005), was used to measure the amount of total carotene in the samples. Hexane, acetonitrile, and ethanol were used to extract the total carotene in a ratio of 50:25:25 (*v:v:v*). About 50 mL of the solvent combination was used to extract 1 mL of the sample. To extract the carotenoids, the liquid was swirled for 15 min in the dark on a magnetic stirring plate. To achieve phase separation, 3 mL of distilled water was added to the mixture and agitated for an additional 5 min. Hexane was used as a blank to measure the absorbance of the filtered hydrophobic phase at 450 nm for total carotenoids. The total carotene was calculated using the formula:

Calculation:

$$\text{Total carotene}\;\left(\mathrm{mg}/100\;g\right)=\frac{\text{Absorbance}\times V\times D\times 100}{W\times Y}$$

Absorbance (450 nm). V = Total volume of extract; D = Dilution factor; W = Weight; Y = Percentage of dry matter content of the sample.

### 2.8 Color determination

Using a Minolta colorimeter (Chroma meter CR‐400C, Konica Minolta, Osaka, Japan) and the CIElab scale L* a* b*, the color of marula fruit juices was determined (Sharma, 2022). The colorimeter was calibrated using a white tile provided by the manufacturer (CIE L* = 96.63, a* = 0.22, b* = 2.28). Petri dishes were filled with the juice sample. Three different (randomly selected) locations where the color of the flour was measured were each averaged. L*, a*, and b* values were used to estimate the hue value (H*), color saturation/treatment intensity (Chroma, C*), and the overall color difference (E*), which were then contrasted with those of the control. All these parameters were calculated using the equations below:

$$\text{Chroma},\left(C*\right)=\sqrt{a^{2}+b^{2}}\;\text{and}\;\mathrm{hue}\;\text{angle}\;\left(H*\right)=\left(b/a\right)$$

$$\text{Total color difference}\;\left(\mathrm{\Delta E}*\right)=\sqrt{{\left(\mathrm{\Delta L}*\right)}^{2}+{\left(\mathrm{\Delta a}*\right)}^{2}+{\left(\mathrm{\Delta b}*\right)}^{2}}$$

Differences of L*, a*, and b* [Equations (1–3)] were used to calculate the changes in different color attributes of samples.

(2) $$\text{∆}L^{*}=L^{*}–L$$

(3) $$\text{∆}a^{*}=a^{*}–a$$

(4) $$\text{∆}b^{*}=b^{*}–b$$

where *L*, *a*, *and b* is color component values of control. The following values were used to determine if the total color difference was visually obvious (Baixauli et al., 2008).

∆E* < 1 = color differences are not obvious to the human eye
1 < ∆E* < 3 = color differences are not appreciated by the human eye
∆E* > 3 = color differences are obvious to the human eye.

### 2.9 Sensory evaluation

A sensory panel of 15 students was chosen at random based on their consumption of various commercially available juices. Before introducing marula fruit juices, they were trained with different blank‐label juices and a mixture of various commercially sold juices to hedonically evaluate the sensory characteristics of the juices. All samples were labeled at random. Each sample was evaluated alphabetically by the panelists for taste, flavor, color, mouthfeel, and overall impression on a 9‐point hedonic scale ranging from 1 for extremely dislike to 9 for like extremely.

### 2.10 Statistical analysis

All analyses were carried out in triplicate. IBM SPSS 25.0 was used to analyze the data. To determine significant differences between the means, ANOVA (one‐way) was used, and the means were separated using the least significant difference (LSD).

## 3 RESULTS AND DISCUSSION

### 3.1 Physicochemical properties of the Marula fruit juice

Table 1 shows the physicochemical properties of the marula fruit juice extracted from steamed and boiled marula fruit. The pH of the control marula fruit juice (raw, unprocessed juice) (4.01) was the least significantly (*p* ≥ .5) different from that of the boiled and steamed marula fruit (4.56 and 4.65, respectively). The results also revealed that steaming significantly increased the juice yield from marula fruits when compared to the raw, untreated fruit (by 108‐fold), but did not significantly (*p* ≥ .5) differ from the yield of boiled fruits. The total soluble sugar of the control sample and marula fruit juice extracted from steamed and boiled marula fruit showed no significant difference (*p* > .05).

**TABLE 1:** Physicochemical properties of marula fruit juice extracted from steamed and boiled marula fruit.

Samples	pH	Yield (%)	TSS (°brix)	TTA (% citric acid)	Vit C (mg/100 mL)
A	4.01^a^ ± 0.1	1.86^a^ ± 0.1	11.23^a^ ± 0.4	3.71^b^ ± 0.5	95.11^c^ ± 0.3
B	4.56^b^ ± 0.1	3.57^b^ ± 0.2	11.70^a^ ± 0.2	3.19^a^ ± 0.4	60.03^a^ ± 0.3
C	4.65^b^ ± 0.1	3.88^b^ ± 0.5	11.87^a^ ± 0.1	3.21^a^ ± 0.3	75.67^b^ ± 0.2

*Note*: Mean ± SD is from three different measurements. Values with different superscripts on the same row are significantly different (*p* > .05). Means in a row with different superscripts are significantly different (*p* > .05) from each other using the least significant difference (LSD).

Keys: A is marula juice extracted from the unprocessed marula fruit. B is marula fruit juice extracted from the boiled marula fruit. C is marula fruit juice extracted from the steamed marula fruit

Abbreviations: TSS, Total Soluble Solid; TTA, Total Titratable acidity.

The titratable acidity (TTA) of the marula fruit juice varied (Table 1). The control sample set (unprocessed marula fruit) had the highest TTA, approximately 16%, which was significantly (*p* > .05) higher than both the boiled and steamed samples. This was accompanied by a significant (*p* > .05) decrease in the vitamin C content, with the boiled and steamed marula fruits having 16% and 58% lower vitamin C contents, respectively, than the control.

The difference in the pH, percentage yield, and total soluble sugar may be attributed to the effects of boiling and steaming marula fruit before the extraction of the juice. The thermal processing techniques of boiling or steaming before extraction can alter the physical composition of the fruit by breaking down certain rigid structures, thus leading to an increased release of juice and an elevated yield of fruit juice. The slight increase in pH may be attributed to the reduction in citric acid present in the marula fruit juice. The increase in the TSS of the marula fruit juice may also be attributed to the release of bound sucrose from the hard cells of the marula fruit which were softened by boiling and steaming the marula fruit before juicing. These results are in line with the report of Legodi et al. (2022), who investigated the conversion of marula fruit juice to wine by spontaneous fermentation.

The decrease in the vitamin C content of marula fruit juice extracted from boiled marula fruit may be attributed to the effects of boiling the fruit juice. Vitamin C is a water‐soluble vitamin that may leach out during boiling (Lathrop & Leung, 1980; Selman & Rolfe, 1982). Also, since vitamin C is heat labile, and the two processes (boiling and steaming) required both heat and water, the boiling and steaming may be responsible for the decrease in the amount of vitamin C in the marula fruit juice. The level of vitamin C degradation in boiled marula fruit juice was higher than that of steamed marula fruit juice. The higher degradation may be attributed to direct contact of the fruit (marula) with the boiling water, which has two negative effects on the vitamin C content of the juice. The first negative effects were the dissolution of vitamin C in water, and the second negative effect was the denaturation of vitamin C due to boiling temperature since vitamin C is heat labile and water soluble, resulting in leaching into the water (Lathrop & Leung, 1980; Selman & Rolfe, 1982). The single negative effect of steaming may be due to the steam temperature, which will degrade the ascorbic acid to dehydroascorbic acid (Mieszczakowska‐Frąc et al., 2021). This is in line with the report of Sharma (2022), who reported a decrease in the vitamin C content of fruit juice when the fruit was steamed before extraction of the juice.

### 3.2 The total phenolic content, antioxidant activities, and total carotene of marula fruit juice

Steamed marula fruit was revealed to have the highest total phenolic content (TPC), significantly (*p* > .05) higher than the control and the boiled fruit (by 688% and 121%, respectively) (Table 2). The highest value of TPC recorded in marula fruit juice extracted from steamed marula fruit may be attributed to the effects of steaming on the marula fruit. Steaming softens the fibers and other cells of fruits, increasing the extractability of both the juice and phenolic compounds that are present in the fruit (Mrabet et al., 2019). Therefore, the low extractability of phenolic compounds in the control could be attributed to the complexity of the fruit matrix, which reduces the extraction of the phenolic compounds. The significantly (*p* > .05) lower TPC observed in the boiled juice as compared to the steamed juice could be attributed to the leaching of water‐soluble phenolic compounds into the boiling water. This result is similar to Saikia and Mahanta (2013), who reported the effects of steaming, boiling, and microwaving on the total phenolic and antioxidant activities of some fruits and vegetables.

**TABLE 2:** Total phenolic content, antioxidant activities, and beta carotene content of marula fruit juice extracted from steamed and boiled marula fruits.

Samples	TPC (μL CE/mg)	ABTS (μmol TE/mg)	DPPH (μmol TE/mg)	Total carotene (mg/100 g)
A	96.42^a^ ± 1.9	58.75^a^ ± 0.6	50.16^a^ ± 0.8	172.24^a^ ± 1.2
B	342.16^b^ ± 1.7	223.13^b^ ± 1.2	297.58^b^ ± 1.4	178.65^b^ ± 1.6
C	756.26^c^ ± 1.4	672.51^c^ ± 1.4	442.91^c^ ± 1.2	184.77^c^ ± 1.7

*Note*: Values are means of triplicates ± standard deviations. Means in a row with different superscripts are significantly different (*p* > .05) from each other using the least significant difference (LSD).

Keys: A is marula juice extracted from the unprocessed marula fruit. B is marula fruit juice extracted from the boiled marula fruit. C is marula fruit juice extracted from the steamed marula fruit.

Abbreviations: CE, catechin equivalents; TE, Trolox equivalents; TPC, Total phenolic content.

The antioxidant activities of the marula fruit juice (ABTS+ and DPPH) followed the same trends. Consistently, the control sample showed the lowest antioxidant capacity against ABTS+ and DPPH when compared to the steamed and boiled samples. Steamed samples showed significantly (*p* > .05) higher ABTS and DPPH when compared to the control and boiled juices (by 11 and 9 times, and 3 and 1.5 times, respectively). The significantly (*p* > .05) lower radical scavenging activity was reported in the control for the total phenolic content of the juice. Phenolic compounds have been implicated in the scavenging of free radicals, and thus a decrease in phenolic content will reflect in a decrease in the radical scavenging properties (Dykes & Rooney, 2006; Shahidi et al., 1992; Shahidi & Zhong, 2015). The higher antioxidant activities recorded for the boiled and steamed marula fruit juice may be directly related to the number of phenolic compounds present in them. Rekha et al. (2012) reported that the scavenging power of fruit juice may be directly proportional to the total phenolic content of such fruit juice. This result is in synergy with the findings of Saikia and Mahanta (2013), who reported an increase in the antioxidant activities of steamed fruits and vegetables.

The total carotene of the marula fruit juice was higher in steamed marula fruit juice than in boiled marula fruit juice (Table 2). Boiling and steaming have positive effects on the marula fruit juice. There was a slight increase in the total carotene content of the marula fruit juice when compared with the control sample. There is a significant difference (*p* > .05) among the three samples. The control sample has the least total carotene content (172.24 mg/100 g), followed by boiled marula fruit juice (178.65 mg/100 g) and steamed marula fruit juice (184.77 mg/100 g). The increase in the total carotene of the boiled and steamed marula fruit juice may be attributed to the effects of boiling and steaming the cells and fiber of the fruit, which may lead to increased ease of extraction of the total carotene content (Jaramillo‐Flores et al., 2003; Miglio et al., 2008; Reis et al., 2010). The potential for steam to permeate the cellular and fiber structures of the marula fruit matrix may lead to an increased concentration of total carotene in the extracted juice (Miglio et al., 2008; Pataro et al., 2018; Reis et al., 2010). Carotene (β‐carotene) is known as pro‐vitamin A which can be converted into vitamin A when the body lacks the vitamin (Vit. A). Beta carotene has a positive impact on cell and tissue growth, delays the onset of aging, and bolsters the immune system against diseases. Additionally, it has been demonstrated to maintain the health and proper functioning of the eyes, skin, nails, and hair (Arumugam et al., 2021). Steaming marula fruit before juicing increases the total carotene extracted from the juice.

### 3.3 Color of the marula fruit juice extracted from boiled and steamed marula fruit juice

The results of the color of the marula fruit juice samples are shown in Table 3. The control samples (marula fruit juice extracted from raw marula fruit) have lightness (L) (54.17) similar to marula fruit juice extracted from steamed marula fruit (58.32). The lightness of the marula fruit juice extracted from boiled marula fruit was the least (42.14). There was a significant difference (*p* > .05) in the lightness (L) of the marula fruit juice sample in this study. The “a” value, which is the measure of reddishness/greenishness of the food sample, showed that marula fruit juice contains a very small amount of red/green color. There was a significant difference at *p* > .05 in the “a” value of all the samples. Marula fruit juice extracted from steamed marula fruit has the highest “a” value (3.65), followed by the control sample (3.18). The marula fruit juice extracted from boiled marula fruit has the least value (2.55). This may be because of boiling the marula fruit juice.

**TABLE 3:** Color of steamed and boiled marula fruit juice extracted from steamed and boiled marula fruits.

Samples	L	a	b	∆E	Chroma
A	54.17^b^ ± 1.73	3.18^b^ ± 0.29	13.26^b^ ± 0.90	43.96^b^ ± 1.73	13.64^b^ ± 0.22
B	42.14^a^ ± 0.52	2.55^a^ ± 0.06	10.24^a^ ± 0.13	55.12^c^ ± 0.52	10.55^a^ ± 0.31
C	58.32^c^ ± 0.44	3.65^c^ ± 0.43	13.68^b^ ± 0.91	40.12^a^ ± 0.23	14.16^c^ ± 0.13

*Note*: Mean ± SD is from three different measurements. Values with different superscripts on the same row are significantly different (*p* > .05). Means in a row with different superscripts are significantly different (*p* > .05) from each other using the least significant difference (LSD).

Keys: A is marula juice extracted from the unprocessed marula fruit. B is marula fruit juice extracted from the boiled marula fruit. C is marula fruit juice extracted from the steamed marula fruit.

The yellowness or blueness of the food sample, which is designated “b” for the marula fruit juice, showed no significant difference (*p* < .05) in values for control samples (13.26) and marula fruit juice extracted from steamed marula fruit (13.68). Marula fruit juice extracted from boiled marula fruit has the least value for “b” which is 10.24. The intensity of the fruit juice color, which is the chroma, showed that marula fruit juice extracted from steamed marula fruit has the highest value (14.16), followed by the control (marula fruit juice extracted from raw marula fruit) at 10.55. The lowest chroma value was recorded for marula fruit juice extracted from the boiled marula fruit.

The highest intensity recorded for the marula fruit juice extracted from steamed marula fruit may be attributed to more of the marula fruit being extracted, which may increase the intensity of the color of the fruit juice (marula). The total color difference (ΔE) showed that all the samples' colors are visible. They were all greater than 3 (>3). The marula fruit juice extracted from the boiled marula fruit has the highest value (55.12), followed by the control sample (43.96) and the marula fruit juice extracted from steamed marula fruit (40.12).

The negative effects of boiling and steaming marula fruit before juicing may be the reason for the difference in the fruit juice color. The boiling of marula fruit before juice extraction may affect the lightness of the extracted juice, thereby reducing the “L.” Steaming is not expected to influence the lightness of the extracted juice since the marula fruit is not in direct contact with water but will help to soften the cell wall for easy extraction of the juice. The color result of the control sample was expected because the marula fruit juice was washed, and the juice was extracted. The highest color intensity value (chroma) recorded for marula juice extracted from steamed marula fruit was expected because of the processing of the marula fruit before extraction. Steaming may help in breaking carotenoid–protein complexes, which may increase the amount of carotenoid in the fruit juice. Also, the reduction in the lightness of the marula fruit juice extracted from boiled marula fruit may be attributed to the browning between amino acids and reducing sugars in the fruit juice. The results of this study were similar to those reported by Yusuf et al. (2023), who reported a decrease in the yellowness of carrot juice processed under high pressure.

### 3.4 Sensory properties of the marula fruit juice extracted from boiled and steamed marula fruits

**FIGURE 1:**
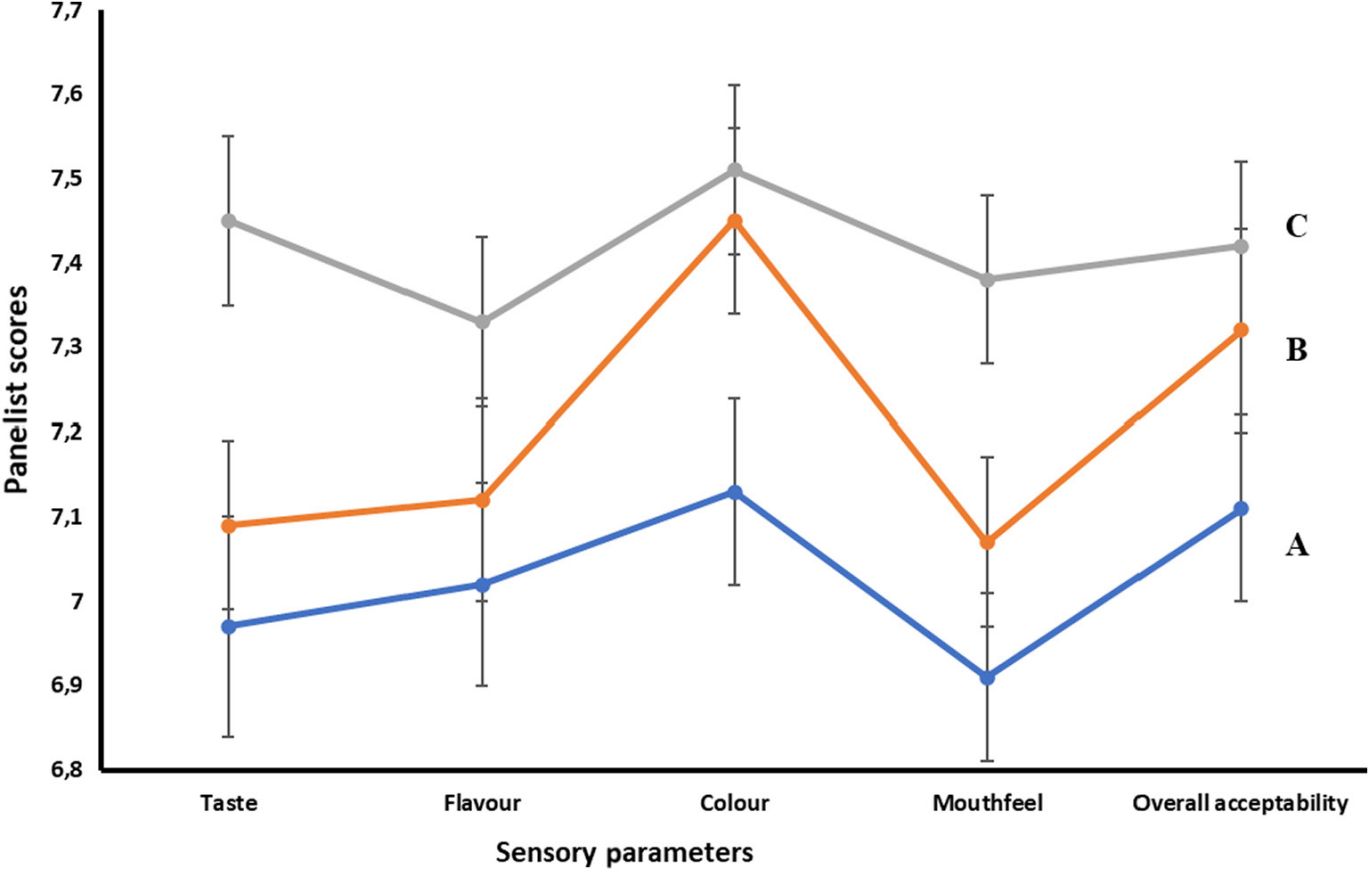
Sensory evaluation of marula fruit juice extracted from steamed and boiled marula fruit. Keys: A is marula juice extracted from unprocessed marula fruit. B is marula fruit juice extracted from boiled marula fruit. C is Marula fruit juice extracted from steamed marula fruit.

The sensory properties of the marula fruit juice are shown in Figure 1. The marula fruit juice was tested on taste, flavor, aroma, mouthfeel, and overall acceptability. The control sample (marula fruit juice extracted from raw marula fruit) scored lower in all the parameters (taste = 6.96, flavor = 7.02, color = 7.13, mouthfeel = 6.91, and overall acceptability = 7.10). The marula juice extracted from boiled marula fruit was scored closer to the control sample (taste = 7.09, flavor = 7.12, color = 7.45, mouthfeel = 7.07, and overall acceptability = 7.32). The marula fruit juice extracted from the steamed marula fruit has the highest score in all the sensory parameters measured (taste = 7.45, flavor = 7.33, color = 7.51, mouthfeel = 7.38, and overall acceptability = 7.42).

Although the panelists were selected based on how regularly they consumed locally extracted juice from local fruits, their score showed that steaming marula fruit before juice extraction improved the taste, flavor, color, mouthfeel, and overall acceptability. This score supported the results we have on the color determination of the marula fruit juice sample (Table 3). The results showed that marula fruit juice from steamed marula fruit had the highest lightness (L) and chroma (intensity) values.

The scores given to each sample may be attributed to the processing (boiling and steaming) that the marula fruit underwent before juicing. Steaming fruits before juice extraction is reported to improve the flavor of the juice (Rios‐Romero et al., 2021), since some trapped flavor compounds may be released during steaming. The released flavor compounds may have positive effects on the taste and color of the juice (marula fruit juice). Boiling, on the other hand, may have similar effects on the flavor compounds but not as significant as those of steaming (reference). Boiling and steaming the marula fruit juice may be responsible for the mouthfeel score and overall acceptability. Since boiling and steaming cause rupture of fruit cells and softening of fruit fiber, more juice may be released during extraction, which the panelists may consider good for the high score given to the fruit juice samples.

**FIGURE 2:**
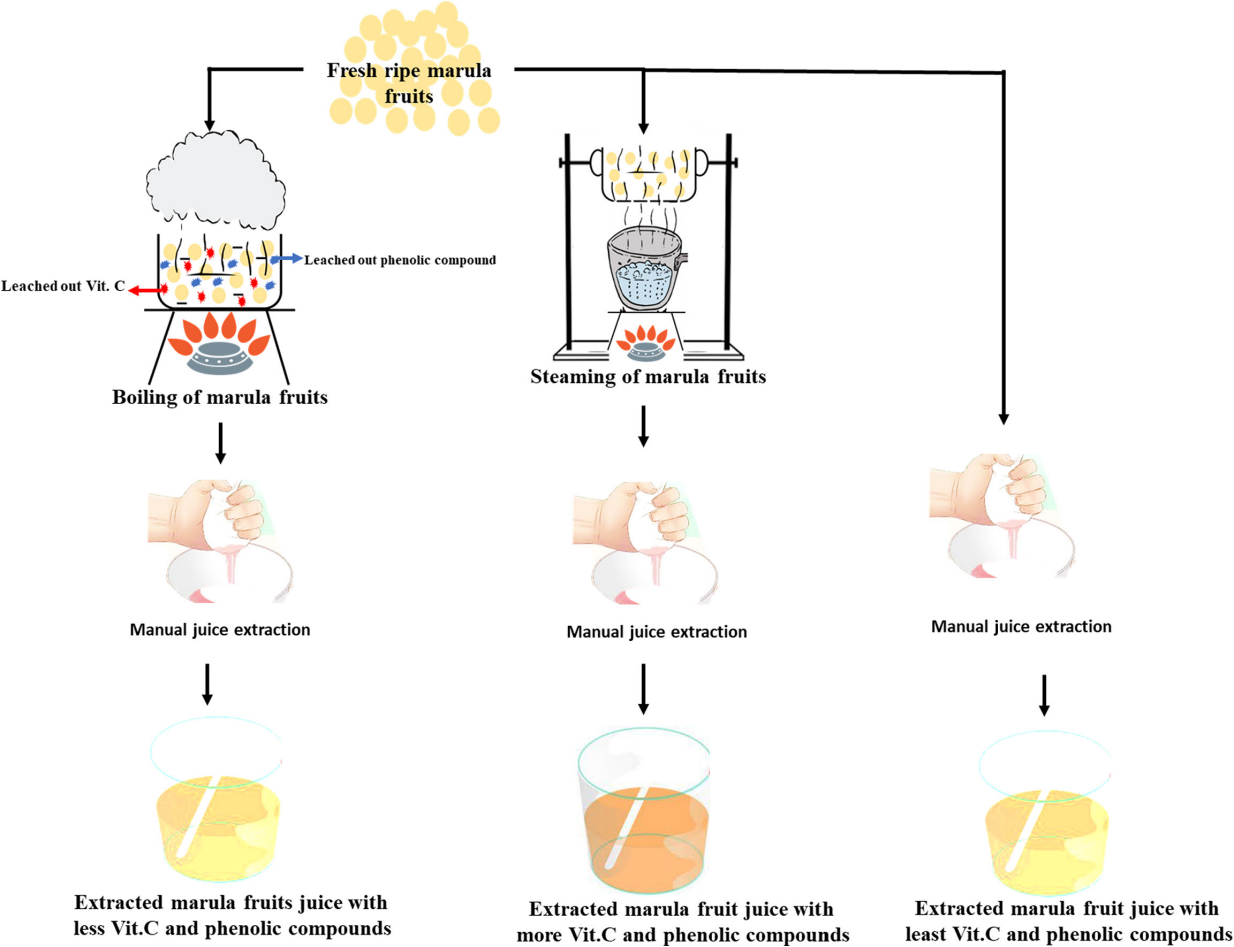
Schematic diagram of the boiling and steaming of marula fruit before juice extraction and the effects of each processing method on the quality of the marula fruit juice.

Figure 2 shows the schematic diagram of the findings in this study. Steaming marula fruit as a unit operation to increase the juice yield could serve as an alternative to the already widely practiced unit operation of boiling the marula fruit. This study has also revealed that steaming marula fruit before juicing (extracting juice) produces a nutritionally superior fruit juice when compared to boiling. Additionally, steamed juice shows higher bioactive phenolic content and antioxidant radical scavenging capacity when compared to its boiled or control counterparts. The color of the juice (marula fruit juice) also was in favor of steaming, as were the sensory parameters measured by the panelist. To get the best nutritional, antioxidant, and sensory parameters from marula fruit juice, steaming the fruit may be the best way to go.

## 4 CONCLUSION

In this study, it can be concluded that marula fruit juice extracted from steamed marula fruit have better physicochemical properties and antioxidant activities than marula fruit juice extracted from boiled marula fruit. The yield of the marula juice also from the three samples shows that steaming marula fruit before juicing is better than boiling and raw. Although boiling marula fruit before juicing may be encouraged rather than juicing raw marula fruits, boiling is detrimental to the vitamin C and total carotene content of the resulting marula juice. In advising the local processor of marula fruit, either juice or wine, steaming the marula fruit will give a better yield with a higher nutritional composition than raw and boiling.

## AUTHOR CONTRIBUTIONS

**Mokoena Z. Dorothy:** Conceptualization (supporting); data curation (supporting). **Terence N. Suinyuy:** Formal analysis (supporting); investigation (supporting). **John Lubaale:** Formal analysis (supporting); writing – original draft (supporting). **Peter O. Bamidele:** Conceptualization (lead); methodology (lead); writing – original draft (lead); writing – review and editing (lead).

## CONFLICT OF INTEREST STATEMENT

The authors confirm that there is no conflict of interest whatsoever with regard to this manuscript.

## Data Availability

The data that support the findings of this study are available on request from the corresponding author.
